# Enhanced Leaching of Lepidolite by Acidophilic Microorganisms Under Mechanical Activation

**DOI:** 10.3390/microorganisms13020415

**Published:** 2025-02-13

**Authors:** Jingna Li, Mengyuan Wang, Ruiyong Zhang, Hongchang Liu, Shiyun Huang, Yang Liu, Rui Liao, Arevik Vardanyan, Jinlan Xia, Jun Wang

**Affiliations:** 1 School of Minerals Processing and Bioengineering, Central South University, Changsha 410083, China; li_jingna@csu.edu.cn (J.L.); mengyuanwang@csu.edu.cn (M.W.); hsy2023@csu.edu.cn (S.H.); liuyang_feiyang@163.com (Y.L.); csuliaorui@163.com (R.L.); jlxia@csu.edu.cn (J.X.); wjwq2000@126.com (J.W.); 2 Key Laboratory of Advanced Marine Materials, Key Laboratory of Marine Environmental Corrosion and Bio-Fouling, Institute of Oceanology, Chinese Academy of Sciences, Qingdao 266071, China; ruiyong.zhang@qdio.ac.cn; 3 Key Laboratory of Biometallurgy of Ministry of Education, Central South University, Changsha 410083, China; 4 Department of Microbiology, SPC “Armbiotechnology” of the National Academy of Sciences of Armenia, 14 Gyurjyan str., Yerevan 0056, Armenia; arevik.vardanyan@asnet.am

**Keywords:** bioleaching, lepidolite, acidophilic microorganisms, mechanical activation

## Abstract

In recent years, mechanical activation technology has been extensively applied as a pretreatment process to increase the leaching efficiency in hydrometallurgical mineral processing. However, studies on its application in the lepidolite bioleaching process are limited. Therefore, the effects of mechanical activation on lithium extraction by an acidophilic iron/sulfur-oxidizing microbial community under different nutrient conditions were evaluated in this study. The solution behavior, phase morphology, and compositional evolution, and microbial community structure succession under eutrophic conditions with exogenous pyrite as the energy substrate and oligotrophic conditions, were investigated. The results revealed that mechanical activation significantly influences the microbial community structure and the interrelationship between microbial activity and mineral phase decomposition and transformation by altering the physical and chemical properties of lepidolite. The best leaching effect was observed in the eutrophic bioleaching groups, followed by the oligotrophic groups at all mechanical activation times. Notably, at a rotation speed of 200 r/min, a material-to-ball mass ratio of 1:20, and an activation time of 150 min, the maximum leaching rates of lithium under eutrophic and oligotrophic conditions were 24.9% and 20.8%, respectively, which were 20.0% and 17.9% higher than those of the nonactivated group. The phase and composition analyses indicated that the dissolution of lithium silicate minerals occurs through a combination of protic acid corrosion, the complexation/electrostatic interactions of extracellular polymeric substances, and the complexation of secondary minerals. These results indicate that the leaching effect is closely related to the pretreatment of mechanical activation, the energy substrates, and the microbial community structure, and this has important reference value for the optimization of the bioleaching process of lepidolite.

## 1. Introduction

Owing to the rapid development of the new energy industry, the importance of lithium has become increasingly prominent [1]. Lithium resources are widely distributed mainly in the form of saltwater and hard rock lithium ores, and most of these resources are brine lithium resources. At present, the composition of brine resources is complex, and the lithium content is low; lithium extracted from brine cannot meet market demand [2]. Therefore, extracting lithium from hard rock lithium ores is currently the primary method. Traditional lithium extraction from ore has several challenges in terms of production costs and environmental risks, owing to the high concentration of acid/alkali, high-temperature roasting, and complex purification processes. Therefore, the development of efficient and clean lithium extraction processes from ore and the comprehensive utilization of resources are inevitable trends in this field [3].

Compared with traditional lithium extraction processes, bioleaching has the advantages of low cost, high efficiency, and environmental friendliness, and it is one of the most promising technologies for processing low-grade and refractory lithium mineral resources. Despite the numerous advantages of lithium bioleaching, most existing applications are in the field of recycling lithium from lithium-ion batteries, and research on the bioleaching of lithium from hard rock ores is scarce. Rezza et al. [4] first used the isolated heterotrophic microorganisms *Penicillium purpurea*, *Aspergillus niger*, and *Rhodotorula mucilaginosa* to extract lithium from spodumene by bioleaching and demonstrated that microorganisms play a certain role in the extraction and accumulation of lithium. During the 30-day bioleaching process, the concentrations of Li in the leachate of these three microorganisms were 1.06 mg/L, 0.5 mg/L, and 0.37 mg/L, respectively. In media with limited concentrations of Mg^2+^, Fe^2+^, and K^+^, the concentrations of Li in the biological leachates were 1.26 mg/L, 1.53 mg/L, and 0.75 mg/L, respectively, which was attributed to the adaptation of microorganisms to low-nutrient environments. Since then, Marcinčáková et al. [5] used the autotrophic bacteria *Acidithiobacillus ferrooxidans* and heterotrophic *Rhodotorula rubra* to extract lithium from lithium-rich lepidolite. However, only 11 mg of lithium was extracted from 1 kg of lepidolite, which proved that the combination of autotrophic bacteria and heterotrophic yeast had no significant effect on lithium leaching. Later, Reichel et al. [6] used sulfur-oxidizing microorganisms to recover lithium from lepidolite, and the leaching rate reached 26% after 54 days. In addition, Sedlakova-Kadukova et al. [7] studied the extraction of lithium from lepidolite using three different biological groups, i.e., *Acidithiobacillus ferrooxidans* and *Acidithiobacillus thiooxidans*, the heterotrophic fungus *Aspergillus niger*, and the heterotrophic yeast *Rhodotorula mucilaginosa*. After 41 days of leaching, the leaching rates of lithium were 8.8%, 0.2%, and 1.1%, respectively. These results show that the traditional bioleaching process of lithium ore has yielded poor results and that the biological extraction mechanism of lithium ores lacks detailed understanding.

Mechanical activation is considered one of the most important pretreatment methods that affect lithium ore solid-phase leachability [8]. Mechanical activation has been confirmed to not only reduce the particle size and increase the specific surface area but also disorder the mineral crystal structure [9]. Lepidolite, a typical lithium ore, exhibits extremely stable chemical inertia due to the interaction of particles inside the crystal. Through mechanical activation, the crystal structure of lepidolite is destroyed, thereby allowing the ore particles to easily participate in chemical reactions in a high-energy activated state. Vieceli et al. [10] reported that after activation of the lepidolite mineral for 10 min and 30 min and digestion at 165 °C for 4 h at an acid concentration ratio of 650 g/kg, the lithium leaching rates reached 82% and 87%, respectively, which confirmed that mechanical activation can improve the reactivity of lepidolite. He et al. [11] reported that the leaching rate of lithium from lepidolite can reach 99.1% under optimal conditions (a mass ratio of mineral to K_2_SO_4_ of 5:1, a ball mill speed of 500 r/min, a ball-to-powder mass ratio of 20:1, a ball milling time of 3 h, a sulfuric acid concentration of 15 vol%, a liquid-to-solid ratio of 4 L/g, a reaction temperature of 80 °C, and a leaching stirring rate of 200 r/min). These results confirm the positive effect of mechanical activation on lithium recovery. However, the recovery rate of lithium slightly decreased to approximately 77% when the mechanical activation duration was extended to 45 min [10]. This suggests that while optimal mechanical activation can significantly increase the leaching efficiency of lithium, excessive activation may lead to adverse effects. In addition, the effects of mechanical activation on biooxidation and gold extraction reported by Beiranvand et al. [12] indicated that the production of ultrafine particles or toxic reactive oxygen species by mechanical activation has a negative effect on the bioleaching efficiency of gold.

Both mechanical activation and biological leaching have been applied individually in the process of extracting lithium from lepidolite. However, there are certain limitations to the application of a single lithium extraction process. The combined application of these two novel processes may hold great potential for extracting lithium from lithium silicate minerals [7]. Therefore, this work studied the effect of mechanical activation pretreatment on the bioleaching of lepidolite using an acidophilic microbial community and explored the effect and mechanism of mechanical activation on the extraction of lithium from lepidolite under both eutrophic (with the addition of pyrite) and oligotrophic (without the addition of pyrite) conditions. The acidophilic microbial community that was used is enriched from the sulfide mining area and relies on reductive iron/sulfur energy substrates to provide electron donors and maintain growth. On the one hand, iron oxidation of acidophilic microorganisms can oxidize Fe^2+^ to high-iron secondary minerals formed by Fe^3+^, which can effectively absorb toxic elements in the environment and reduce the toxicity of the leaching environment; on the other hand, sulfur oxidation can oxidize reductive sulfides or S^0^ to sulfuric acid to maintain the acidic environment of the system. It is hypothesized that under the action of mechanical activation, the acidophilic microbial community employs different mechanisms for the enhanced leaching of lepidolite minerals under eutrophic/oligotrophic conditions. To verify this hypothesis and reveal the enhancing effect of mechanical activation pretreatment on the bioleaching of lepidolite, the changes in the particle size, specific surface area, mineral structure, and morphology of lepidolite under different mechanical activation times were examined, and the surface composition and morphology of the leaching residues, mineral phase evolution, and microbial community shifts in the process of lepidolite bioleaching under eutrophic/oligotrophic conditions were compared. These results will help us understand the biological extraction mechanism of lithium from hard rock ores and broaden the application scope of bioleaching technology.

## 2. Experimental Section

### 2.1. Materials

#### 2.1.1. Strain and Culture Medium

The acidophilic microorganisms used in this study were enriched from the Dabaoshan sulfide mining area and exhibited effective iron/sulfur oxidation after long-term domestication. The original microbial diversity used in the leaching experiment was analyzed via high-throughput 16S rDNA sequencing. The results showed that its composition is mainly chemoautotrophic microbes. The dominant species were unclassified *Leptospirillum* (41.8%), moderately thermophilic acidophilus *Acidithiobacillus caldus* (30.8%), and the chemoheterotrophic acidophilus acid globobacterium *Acidisphaera* sp. PS110 (26.3%). The enriched acidophilic microorganisms were cultured in 9K basal media supplemented with FeS_2_ as an energy substrate. All the reagents were analytically pure, and deionized water was used. The acidophilic microorganisms were domesticated and cultured in 9K media supplemented with 10 g/L lepidolite and 10 g/L pyrite before the formal experiment.

#### 2.1.2. Ore

**Figure 1:**
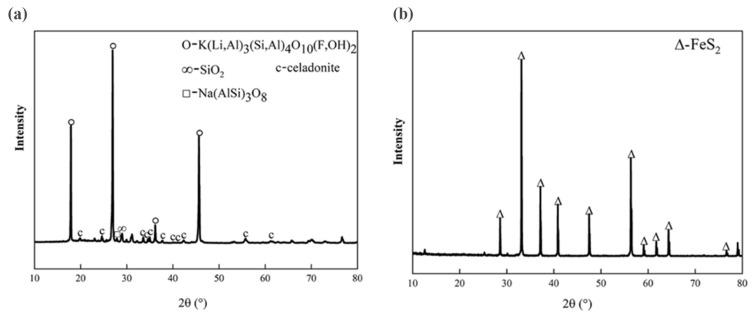
XRD patterns of the pristine (**a**) lepidolite minerals and (**b**) pyrite.

The lepidolite and pyrite used in this experiment were obtained from the School of Minerals Processing and Bioengineering, Central South University, Changsha, China, and were sieved to 400–200 mesh (38–74 μm). The composition of the lepidolite was measured via X-ray fluorescence (XRF, Bruker S6 Jaguar, Bruker (Beijing) Scientific Technology Co., Ltd., Beijing, China) spectrometry. The contents of lithium, aluminum, and iron in the lepidolite were 3.20%, 5.05%, and 0.04%, respectively, as determined via inductively coupled plasma-mass spectrometry (ICP-MS, PerkinElmer NexION 300X, PerkinElmer, Inc., Waltham, MA, USA) (Table S1). X-ray diffraction (XRD, Bruker D8A A25X, Billerica, Germany) analysis of the pristine lepidolite (Figure 1a) revealed that the main minerals were lepidolite ([K(Li,Al)_3_(Si,Al)_4_O_10_(F,OH)_2_]), albite ([Na(AlSi_3_)O_8_]), and quartz (SiO_2_) [13]. The pyrite samples were crushed, milled, and screened before the experiment, and 200–400 mesh samples were selected for follow-up physical and chemical analyses. The results of the XRD analysis (Figure 1b) revealed that the main phase in the sample was pyrite (FeS_2_).

### 2.2. Methods

#### 2.2.1. Mechanical Activation Treatment of the Lepidolite Mineral Samples

Mechanical activation of the lepidolite mineral was performed in a planetary ball mill (Deco Company of China, Guangzhou, China). The mechanical forces applied by this equipment include impact, friction, and compression. The grinding cans (50 mL) and grinding balls are made of stainless steel, and the lepidolite can be highly crushed in a short time by mechanical forces such as impact, friction, and compression in the processes of mechanical activation [11]. Considering the properties of the lepidolite and equipment performance, the rotational speed was controlled at 200 r/min. Based on prior research, the ball-to-material mass ratio was selected as 20:1. According to the preliminary impacts of mechanical activation on the particle size and specific surface area, activation times of 30 min, 60 min, 90 min, 120 min, and 150 min were used to study the effects of mechanical activation on the bioleaching of lepidolite. The activated and nonactivated samples were subjected to the following leaching experiments.

#### 2.2.2. Determination of Particle Size and Specific Surface Area

The particle size distributions and specific surface areas of both the activated and nonactivated lepidolite samples were determined via a laser particle size analyzer manufactured by Malvern, UK. The procedure was as follows: the refractive index and absorbance of the lepidolite sample were set, with water selected as the dispersant. The purity of the device was measured via a background measurement function, followed by a background test using a special beaker containing 600–800 mL of tap water. Prior to sampling, the lepidolite powder was poured into a small beaker, water was added, and the sample was continuously stirred with a pipette to ensure uniformity of the slurry. Simultaneously, the sample was added to the dispersion tank via a pipette. The sample was then subjected to ultrasonication for 2 min at a pump speed of 2000 rpm/min for 5 min before testing.

#### 2.2.3. Bioleaching Experiments

**Figure 2:**
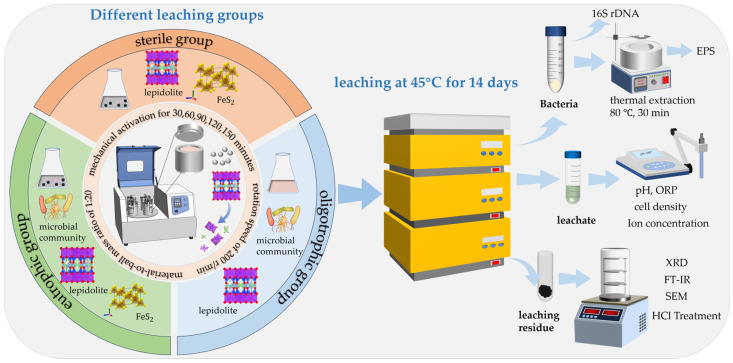
Experimental systems and their corresponding characterization techniques.

Lepidolite leaching experiments were carried out in 250 mL Erlenmeyer flasks. A total of 100 mL of 9K basal medium was used, and the initial pH was adjusted to 2.0 with 1 M sulfuric acid and sterilized at 121 °C for 15 min. To study the bioleaching effect of acidophilic microorganisms on lepidolite under different mechanical activation times (0, 30, 60, 90, 120, and 150 min) and to determine the best duration of mechanical activation, different experimental groups were set up: (i) Eutrophic bioleaching groups involving the addition of 10 g/L lepidolite activated for various durations (0, 30, 60, 90, 120, and 150 min) along with 10 g/L pyrite as an energy substrate. (ii) Oligotrophic bioleaching groups with 10 g/L lepidolite activated for the same duration but without any pyrite. (iii) Sterile control groups without pyrite. The acidophilic microorganisms that grew to the middle logarithmic phase were washed with freshly sterilized 9K basal medium at pH 2.0, centrifuged at 10,000 r/min 2–3 times, and inoculated into all bioleaching groups at an initial density of 5 × 10^7^ cells/mL. The conical flasks were placed in an oscillating incubator (ZQZY-A8) at a constant temperature of 45 °C and an oscillating speed of 180 r/min to study the effects of mechanical activation and nutrient conditions on the bioleaching of lepidolite. The whole leaching experimental period was 14 days. During the experiment, the conical bottle was weighed every day, and the liquid lost due to evaporation was replenished with sterile deionized water. Three parallel experiments were performed for each group. During the course of the experiment, the solution samples were collected every 2 days, and physical and chemical parameters such as the pH, oxidation–reduction potential (ORP), microbial cell concentration, and ion concentration in the leaching solution were determined. The solid leaching residue collected at the end of the bioleaching experiment was reserved for subsequent phase tests and hydrochloric acid treatment. The specific experimental systems and characterization test methods are shown in Figure 2.

#### 2.2.4. Analysis of the Solution Behavior

The pH was monitored by a pH meter (FE28, Mettler Toledo International Co., Ltd., Shanghai, China), and the ORP was measured by a Pt electrode with an Ag/AgCl electrode as the reference (PHSJ-4F, Shanghai INESA Scientific Instruments Co., Ltd., Shanghai, China). The changes in the concentration of the suspended microbial cells during the experiment were determined under an optical microscope (Olympus, CX33, Tokyo Metropolis, Japan) via a hemocytometer. The extracellular polymer was extracted by the thermal extraction method (80 °C, 30 min) and collected. The concentrations of elements in the leaching solution, EPSs, and secondary minerals after leaching were determined via inductively coupled plasma-optical emission spectrometry (ICP-OES; IRIS Intrepid 161 II XSP, Thermo Fisher, Waltham, MA, USA).

#### 2.2.5. Extraction of Extracellular Polymeric Substances

After 14 days of the bioleaching experiments, the solid–liquid mixtures from the bottom of each conical flask system were individually transferred to centrifuge tubes. The mixtures were subsequently centrifuged at 10,000 rpm/min for 10 min to separate the supernatants. The mixtures were subsequently centrifuged at 2000 rpm/min for 3 min to isolate microbial cells from the culture medium and leaching residues. The cells were then washed repeatedly with phosphate-buffered saline and collected. Extracellular polymeric substances (EPSs) from acidophilic microorganisms in each bioleaching system were extracted via a thermal extraction method (heating in a water bath at 80 °C for 30 min); polysaccharides constitute a significant component of EPSs, and their presence was quantified through the chromogenic reaction using the phenol–sulfuric acid method, with absorbance measurements taken at a wavelength of 490 nm to validate the polysaccharide content within the EPS. Additionally, the accumulated lithium-ion concentration in the EPS was determined.

#### 2.2.6. Hydrochloric Acid Treatment of Leaching Residue

Secondary minerals such as jarosite are formed during the oxidation of pyrite by acidophilic microorganisms [14,15], and the lithium ions leached into the solution may enter the lattice of the jarosite or adsorb on the surface of the jarosite. In addition, EPSs produced by microorganisms may adsorb lithium ions in solution via complexation/electrostatic interactions. At the end of the bioleaching experiments, the leaching residues were collected and treated with hydrochloric acid, as shown in Figure S1. After the lepidolite had been leached for 14 days, all the samples were centrifuged for 10 min at a rotational speed of 10,000 r/min, and solid–liquid separation was carried out. The residues were vacuum freeze-dried and treated by acid leaching for 24 h, and the same volume of 1 M HCl was used as the separation supernatant. To calculate the Li recovery efficiency, the Li concentrations in the filtrate and bioleaching residue containing minerals and microorganisms were analyzed.

#### 2.2.7. Calculation of the Lithium Leaching Rate

To calculate the recovery rate of lithium, the ion concentrations in the bioleaching solution, EPSs, and secondary minerals should be considered. The leaching rate of Li was calculated via Equation (1):

(1) $$Leaching\;rate=\frac{C_{S}\times V_{S}+C_{H}\times V_{H}}{C_{F}\times M_{F}}$$

*C_S_* is the total metal Li concentration (mg/L) determined via ICP-OES of the bioleaching filtrate, and *V_S_* is the volume (L) of the bioleaching solution. *C_H_* is the concentration of lithium ions (mg/L) adsorbed by EPSs and secondary minerals, denoted as the difference in the lithium-ion concentration in the supernatant between the bioleaching residue treated with hydrochloric acid and that of the sterile system leaching residue. *V_H_* is the volume (L) of HCl used in the chemical treatment. *C_F_* refers to the content of metallic lithium in lepidolite (mg/L) determined by X-ray fluorescence (XRF), and *M_F_* is the mass (g) of lithium ore powder added to each leaching system. Owing to the difficulty in discerning the source of Li^+^ leached by HCl, whether from lepidolite or secondary minerals, and the metabolic activities of microorganisms promote the entry of more lithium ions into the leachate, which results in a lower lithium content in the bioleaching residues than in the sterile control residues, the actual calculated value of *C_H_* in the formula is less than the theoretical value. Consequently, the actual leaching efficiency in this study is slightly higher than the theoretical value calculated in this paper.

#### 2.2.8. Analysis of the Mineral Phase Composition and Surface Morphology

The mineral phase composition was analyzed via X-ray diffraction (XRD) (Bruker D8A A25X, Billerica, Germany) with a scanning angle ranging from 10° to 80° and a scanning speed of 5°/min. The distribution of elements on the surface of the minerals was analyzed by scanning electron microscopy (SEM; MIRA4 LMH) and X-ray energy spectroscopy (EDS; Oxford AZtecLive Ultim Max 20, Oxford, UK). The solid samples for SEM were observed by sputtering with gold (JEOL, JF-1600, Tokyo, Japan) and were then placed in a scanning electron microscope room. The FT-IR spectra were detected by a Fourier transform spectrometer (Nexus 670, Nicolet, Waltham, MA, USA) in transmittance mode. The FT-IR spectra were recorded from 400 to 4000 cm^−1^ after mixing 4 mg of each sample with 250 mg of KBr and pressing the mixture into a pellet.

#### 2.2.9. 16S Sequencing of Bacterial Communities

Microbial diversity was determined at Beijing Qingke Biotechnology Co. Ltd., Beijing, China, via the Illumina NovaSeq sequencing platform. A small fragment library was constructed for sequencing via the paired-end method. On the basis of the conserved region of the nucleic acid sequence encoding ribosomal RNA (16S region), reads were assembled, filtered, clustered, or denoised, and species annotation and abundance analysis were performed to reveal the microbial community structure of the samples.

## 3. Results and Discussion

### 3.1. The Effects of Mechanical Activation on the Properties of Lepidolite

#### 3.1.1. Analysis of Particle Size and Specific Surface Area

**Figure 3:**
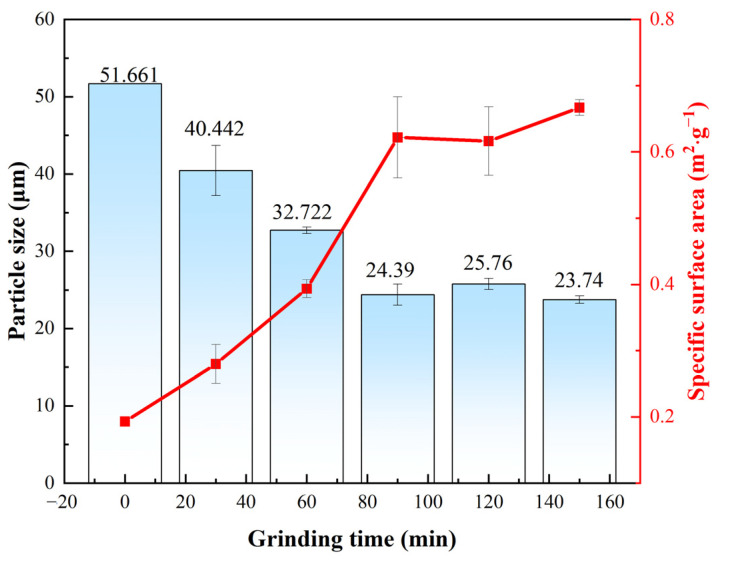
The average particle size and specific surface area of the lepidolite.

The most notable effects of mechanical activation were a reduction in the particle size and an increase in the specific surface area (Figure 3). The changes in the average particle size and specific surface area of the lepidolite with increasing activation time indicated that within 90 min of activation, strong fragmentation rapidly reduces the particle size of the lepidolite, and the average particle size and specific surface area exhibit linear variation. From 90 to 120 min, the trends for the average particle size and specific surface area of the lepidolite reversed compared with those within 90 min. The increase in average particle size and decrease in specific surface area during this period may be attributed to the adverse effects of particle reaggregation due to excessive activation time [10]. Within 90 to 150 min, the average particle size and specific surface area of the lepidolite exhibited minor fluctuations but remained essentially stable, indicating that the mechanical activation had reached equilibrium. Consequently, the mechanical activation time range of 0 to 150 min was selected for subsequent characterization and analysis.

#### 3.1.2. Changes in the Morphology of Lepidolite

**Figure 4:**
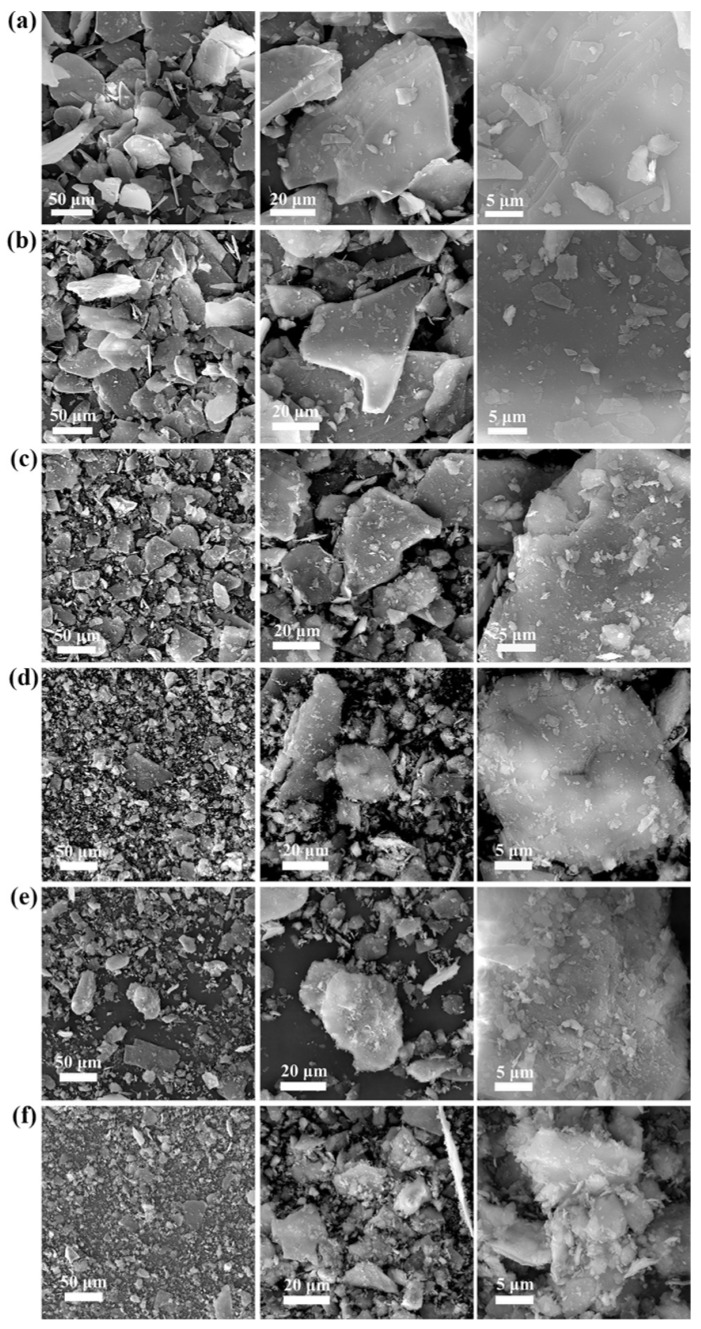
SEM images of pristine lepidolite minerals (**a**), activated lepidolite after 30 min of grinding (**b**), activated lepidolite after 60 min of grinding (**c**), activated lepidolite after 90 min of grinding (**d**), activated lepidolite after 120 min of grinding (**e**), and activated lepidolite after 150 min of grinding (**f**).

The morphological changes in the mineral particles throughout the process were evaluated through SEM analysis of the lepidolite at different activation times (0–150 min) (Figure 4). Nonactivated lepidolite ore typically possesses a complete and distinct multilayer structure (Figure 4a). The primary result of mechanical activation is the fracturing of the mineral particles. After 30 min of mechanical activation, compared with the nonactivated lepidolite, there was only a reduction in particle size, with no significant morphological changes (Figure 4b). The process of high-energy grinding is accompanied by an increase in the particle count and the emergence of unexposed fresh surfaces. After 60 min of activation, the surface of the lepidolite started to display irregular amorphous particles (Figure 4c). Beyond 90 min of activation, in addition to a decrease in the particle size, the stable multilayer structure gradually disappeared, with small particles becoming dominant (Figure 4d). After 120 min of activation, some of the fine particles reunited (Figure 4e), and prolonged grinding promoted such particle composite phenomena [16]. After 150 min of activation, the aggregation of fine particles became more noticeable (Figure 4f), probably because small particles resulting from mechanical activation reduce their own surface area by clumping together, thereby reducing energy and achieving a more stable state [17]. The SEM results largely align with the particle size distribution analysis (Figure 3) assessed by the laser diffraction method.

#### 3.1.3. Analysis of Mineral Composition

**Figure 5:**
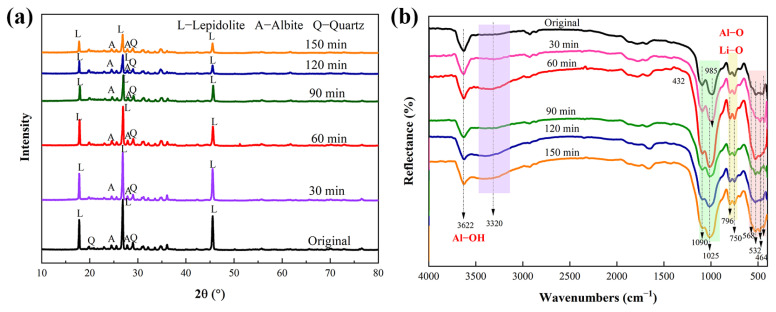
XRD patterns (**a**) and FTIR spectra (**b**) of the lepidolite before and after mechanical activation.

XRD analysis before and after mechanical activation (Figure 5a) revealed that the characteristic diffraction signals of the nonactivated lepidolite were clear and strong. After mechanical activation, there was little change in the peak shape of the lithium silicate ore, and no new diffraction peaks were generated, indicating that no new substances formed during mechanical activation. After 60 min of mechanical activation, there was a downward trend in the characteristic peak of the lepidolite, and after 90 min of activation, the peak intensity significantly decreased.

The FTIR spectra of the activated and nonactivated lepidolite samples are shown in Figure 5b. The FTIR spectrum of the nonactivated lepidolite was very similar to that of the poly-lithium sulfide ore, with a clear peak appearing at 3622 cm^−1^, which is different from the broad peak caused by the presence of hydroxyl groups in water. This peak is the absorption peak of the hydroxyl stretching vibration, attributed to the Al-OH bond in lepidolite [11]. After 60 min of mechanical activation, the transmittance of this absorption peak tended to decrease, and the change after 90 min was more noticeable. In addition, before 90 min of activation, no more significant changes were detected, and there were only minor differences in peak positions. After 90 min of activation, the SiO_4_ stretching vibration band (approximately 1025 cm^−1^) tended to broaden. The relative intensities of the double peaks at 796 and 754 cm^−1^ changed after mechanical activation. The inverted “mountain-shaped” absorption peak in the low-frequency range of 568–464 cm^−1^ is a characteristic peak of muscovite, and it tends to merge into one peak attributed to the destruction of Al-O and Li-O bonds [18]. These results, combined with the XRD results (Figure 5a), indicated that dehydroxylation and amorphization were the main structural changes that occurred during the mechanical activation of the lepidolite.

### 3.2. Solution Behavior During the Leaching of Lepidolite

**Figure 6:**
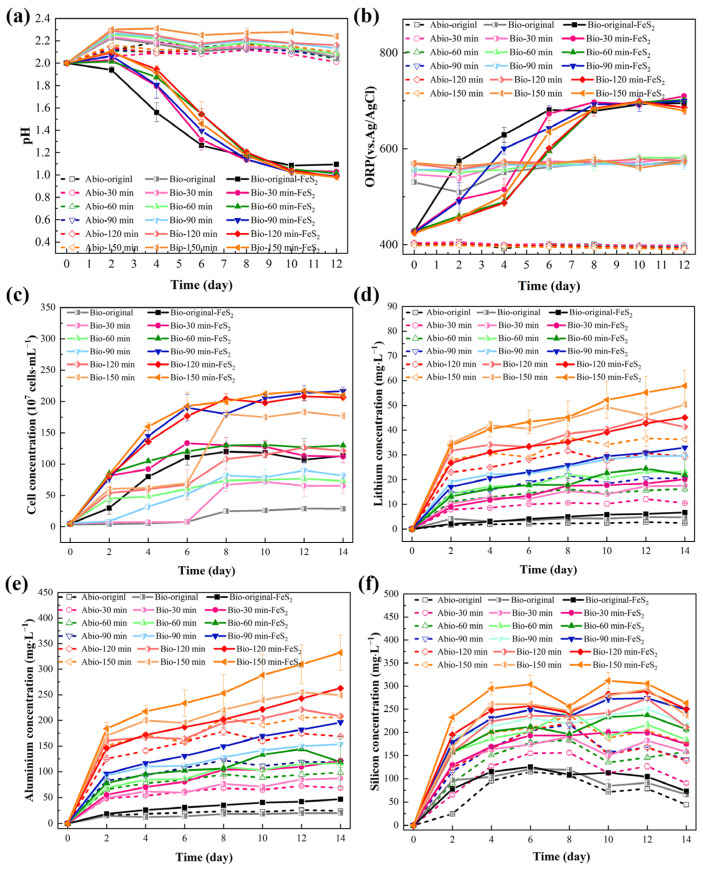
The solution behavior in the leaching groups: (**a**) pH, (**b**) ORP, (**c**) bacterial concentration, (**d**) [Li^+^], (**e**) [Al^T^], and (**f**) [Si^T^].

Nonactivated lepidolite ore and samples with different mechanical activation times were leached individually in the eutrophic, oligotrophic bioleaching, and sterile control groups. In the initial stage of leaching (0–2 days), a slight increase in pH was observed in almost all the groups. (Figure 6a), which can be attributed primarily to the exchange between Li^+^/K^+^ and other ions in lepidolite and H^+^, as well as the acid dissolution reaction of lepidolite, which consumes a small number of protons. The pH increase trend was slowest in the eutrophic groups, and there was no increasing trend throughout the entire experiment in the nonactivated eutrophic groups. This might be explained by the preferential reaction of the microorganisms with pyrite, coupled with a higher rate of acid production than acid consumption by the lepidolite. In all the eutrophic bioleaching groups, the pH decreased, primarily due to the oxidation of pyrite by acidophilic microorganisms, which generated sulfuric acid and provided an abundant source of H^+^ ions (Equations (2) and (3)). Notably, the eutrophic groups containing pyrite were subjected to nonuniform pH changes due to the presence of microbial ferrous oxidation-consuming protons (Equation (4)) and the formation of secondary minerals in the later stage. In contrast, in the oligotrophic bioleaching groups and sterile control groups, the pH of the leachate remained relatively stable at approximately 2.0 throughout the leaching period.

(2) $${FeS}_{2}+{2Fe}^{3+}\to {3Fe}^{2+}+{2S}^{0}$$

(3) $${2S}^{0}+{3O}_{2}+{2H}_{2}O\to {2SO_{4}}^{2-}+{4H}^{+}$$

(4) $${4Fe}^{2+}+{4H}^{+}+O_{2}\to {4Fe}^{3+}+{2H}_{2}O$$

The change in the ORP in the leaching solution was affected by microbial metabolic behavior and reactants (Figure 6b), especially the changes in the concentrations of Fe^2+^ and Fe^3+^. The eutrophic groups, before and after mechanical activation, initially had an ORP of approximately 420 mV, which continuously increased to approximately 700 mV within 0–8 days and then tended to stabilize. Acidophilic microorganisms can oxidize Fe^2+^ to Fe^3+^, resulting in an increase in the [Fe^3+^]/[Fe^2+^] ratio in the solution, which resulted in a rapid increase in the ORP. After 8 days, the concentrations of Fe^2+^ and Fe^3+^ reached a state of equilibrium, thereby maintaining a stable ORP. All oligotrophic groups and sterile control groups exhibited only minimal changes throughout the entire experiment.

During bioleaching in both the eutrophic and oligotrophic groups, there were notable differences in the changes in the free acidophilic microorganism concentration over time (Figure 6c). In all the eutrophic bioleaching groups, there was no significant decrease in the microorganism concentration, indicating that the leaching groups are adaptable and that the oxidation of pyrite can promote the growth of microorganisms [14]. In oligotrophic groups, the variation in microorganism concentration was relatively slower than that in eutrophic groups; however, overall, there was still an increasing trend. This occurred because there is no additional energy substrate in oligotrophic groups, whereas lepidolite contains small amounts of elements such as Fe and S to provide energy for microorganisms. Sedlakova-Kadukova et al. [7] reported that microorganisms may be forced to use the nutrients needed for their life directly in the leaching solution and lepidolite. In addition, the studies of Rezza et al. [4] also showed that metabolites such as gluconic acid, citric acid, and extracellular polymers in the leaching solution are related to bioleaching, and the important factor in enhancing this process may be the adaptation of microorganisms to oligotrophic environments. Notably, the concentration of microorganisms in the solution appeared to be positively correlated with the duration of mechanical activation. After 150 min of activation, the concentration in the oligotrophic leaching groups reached approximately 35 times the initial inoculation volume. This could be attributed to the generation of fresh surfaces from mechanical activation, which provides more contact sites between microorganisms and lepidolite, thereby enhancing the energy availability for microbial growth and metabolism.

In lepidolite, it is predicted that Li is bound between layers of AlO_6_ in an octahedral structure and SiO_4_ in a tetrahedral structure, acting predominantly as a charge supplement to ions. Therefore, the dominant bonding force is predicted to be highly ionic in these crystal structures [19]. Lithium, aluminum, and silicon ions are crucial in the crystal structure of lepidolite. Therefore, the contents of lithium, aluminum, and silicon were detected mainly during the lepidolite leaching process. The trend of [Li^+^] in the solution in the leaching process (Figure 6d) indicated that [Li^+^] was proportional to the duration of mechanical activation. This is because mechanical activation can cause cracks in the ore particles to increase the access of the microbial cells and electron shuttles to the minerals, thereby promoting the leaching of ions from the lepidolite. Moreover, under identical activation durations, the eutrophic groups presented the highest concentration of [Li^+^], followed by the oligotrophic bioleaching groups, whereas the sterile groups presented the least effective lithium leaching capability. The variation trend of [Al^3+^] (Figure 6e) in the solution was consistent with that of [Li^+^]. The concentration changes of silicon were more complex (Figure 6f), which might be attributed to the formation of silicate minerals due to the combination of most metal ions with free silicate ions [16], resulting in a change in the solubility of silicon, as shown in Equation (5), where M stands for metal ions. The silicate minerals subsequently decomposed in the acid-leaching group, and some silicate components were released into the solution.

(5) $$M^{n+}+{mSiO}_{3}^{2-}\to {M(SiO}_{3}{)}_{m}$$

Furthermore, the variations in [Li^+^] within the leachate may be influenced by two main factors. During bioleaching, EPSs are secreted by microorganisms during growth. These entities are typically composed of charged functional groups or macromolecular polymers, such as polysaccharides, proteins, and nucleic acids, which may engage in electrostatic interactions or complexation with Li^+^. In addition, for eutrophic groups, under conditions of high ORP and [Fe^3+^], iron-containing secondary minerals (such as jarosite) adsorb Li^+^ or undergo Li^+^ substitution to embed it into the lattice (Equation (6)).

(6) $$M^{+}+{3Fe}^{3+}+{2SO}_{4}^{2-}+{6H}_{2}O\to {MFe}_{3}{(SO}_{4}{)}_{2}{\left(OH\right)}_{6}+{6H}^{+}$$

where M is a univalent cation, such as K^+^, NH_4_^+^, H_3_O^+^, or Li^+^.

### 3.3. Results of Hydrochloric Acid Treatment of Leaching Residue

**Figure 7:**
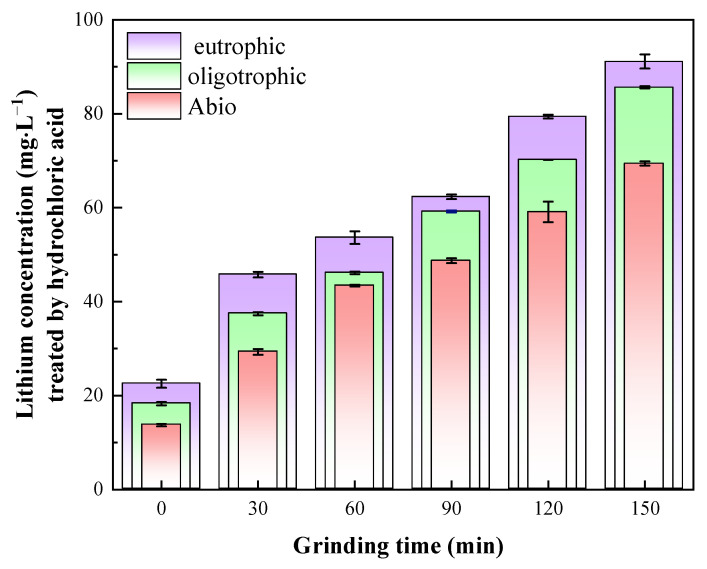
Lithium content in solution after HCl leaching.

To elucidate the actual leaching rate of lithium and the leaching mechanism of lepidolite, the bioleaching residues were further subjected to acid leaching treatment with a hydrochloric acid solution. After the bioleaching residue was leached with dilute hydrochloric acid for 24 h, [Li^+^] was found to be highest in the eutrophic bioleaching group, followed by the oligotrophic group and, finally, the sterile control group. The leaching of lithium ions in the sterile control groups was attributed primarily to the chemical effects of dilute hydrochloric acid, resulting in a relatively low dissolved lithium content during this process. Through thermal extraction, high-temperature treatment was employed to disrupt the cell membranes of acidophilic microorganisms, thereby facilitating the release of EPSs and subsequent isolation of polysaccharides. The extracted polysaccharide derivatives demonstrated a distinct chromogenic reaction with phenol under strongly acidic conditions, yielding characteristic orange–yellow compounds (Figure S2). Spectral analysis revealed a prominent absorbance peak at 490 nm for these compounds, with detailed quantitative data provided in Table S2. ICP-OES analysis confirmed the presence of lithium ions in the EPS-containing supernatant (Table S3), indicating that the lithium ions released in the oligotrophic bioleaching system were also attributed to the dissociation of EPSs under the action of dilute hydrochloric acid, although the EPS content was very small. For the eutrophic bioleaching groups, the release of lithium ions included the chemical effects of dilute hydrochloric acid, EPS dissociation, and the release of Li^+^ adsorbed by secondary minerals. A comprehensive comparison among these three groups revealed significant differences in lithium leaching after hydrochloric acid treatment, indicating that during bioleaching, most of the Li^+^ existed in a dissolved state in the solution, whereas only a small amount was adsorbed by EPSs and secondary minerals. This discovery provides an important basis for our in-depth understanding of the migration and transformation mechanism of Li^+^. Notably, the results in Figure 7 also show that the leaching rates of lithium for all the groups were positively correlated with the mechanical activation time. The leaching residue activated for 150 min had the best leaching effect with dilute hydrochloric acid, and the lithium concentrations of the solutions were 62.33 mg/L, 79.40 mg/L, and 91.13 mg/L. Therefore, to further study the role of the microbial community in bioleaching and the relevant mechanism, the groups with lepidolite activated for 150 min and nonactivated lepidolite were selected for subsequent analyses.

### 3.4. The Mineral Composition and Surface Morphology of the Leaching Residue

#### 3.4.1. SEM Analysis

**Figure 8:**
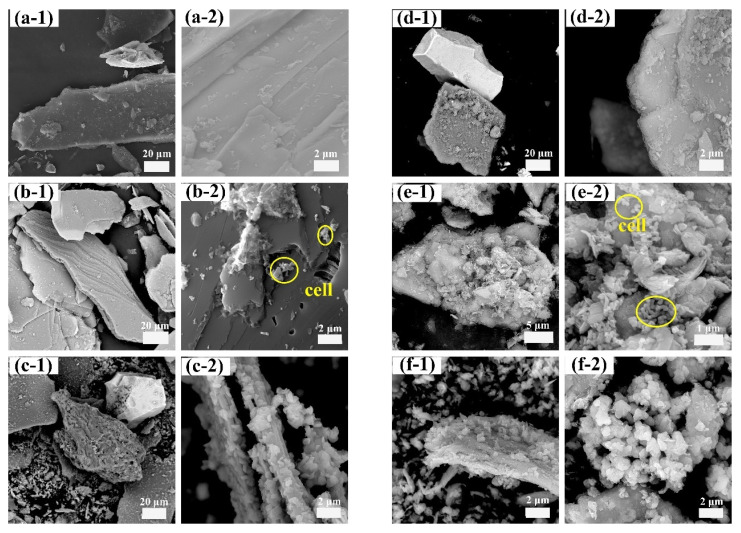
SEM morphology: sterile control group (**a-1**,**a-2**); nonactivated oligotrophic group (**b-1**,**b-2**); nonactivated eutrophic group (**c-1**,**c-2**); sterile control group after activation for 150 min (**d-1**,**d-2**); oligotrophic group after activation for 150 min (**e-1**,**e-2**); and eutrophic group after activation for 150 min (**f-1**,**f-2**). The circles in panels (**b-2**,**e-2**) represent the absorbed cells.

SEM analysis revealed that the morphological and structural characteristics of the leaching residues from the sterile, oligotrophic, and eutrophic systems were comparable after 120 and 150 min of mechanical activation (Figure S2). Consequently, representative samples of the residues from the nonactivated and 150 min mechanically activated lepidolite groups were selected for further analysis of their morphology and elemental distribution. Before activation, after 14 days of leaching in the sterile control groups, the lamellar morphology of the lepidolite was still clearly visible (Figure 8a-1,a-2). When nonactivated, the lamellar morphology of the lepidolite was still clearly visible after 14 days of leaching in the sterile control groups. However, under the influence of microorganisms, the mineral surface underwent significant changes, initially appearing in very fragile parts of the mineral surface (Figure 8b-1,b-2,e-1,e-2). In addition to the appearance of porous structures on the surface of the lepidolite, in the eutrophic groups, the ore particles dissolved during the leaching process and decomposed into smaller particles, which were wrapped around the lepidolite surface and continued to decompose, and the pyrite surface eroded. After 150 min of mechanical activation, the mineral morphology in the sterile control group was still intact (Figure 8d-1,d-2). The mineral changes in the bioleaching groups were more obvious, and bacterial adsorption was observed. Lepidolite is locally loose, fine-grained, porous, and fragmented, which is the result of bacterial action (Figure 8c-1,c-2,f-1,f-2) [20]. Prolonged mechanical activation enables acidophilic microorganisms to penetrate deeper into minerals for more effective direct bioleaching. In addition, after 150 min of mechanical activation, the eutrophic groups developed abundant secondary minerals displaying a unique morphology of loosely aggregated nanoparticles. The mineral formation process was substantially controlled by lepidolite fragments and their associated elemental compositions, directing the crystallization pathway toward jarosite formation (Figure 8f-2). The EDS results (Figure S3) revealed that the mineral phase was predominantly composed of Fe and S, with a minor proportion of K. This elemental composition is consistent with the characteristic chemical signature of jarosite minerals, indicating that mechanical activation might promote the formation of such secondary minerals. These secondary minerals not only adsorb lithium ions but also serve as microbial attachment substrates, thereby further enhancing the microbial bioleaching capacity.

#### 3.4.2. XRD and FTIR Analysis

**Figure 9:**
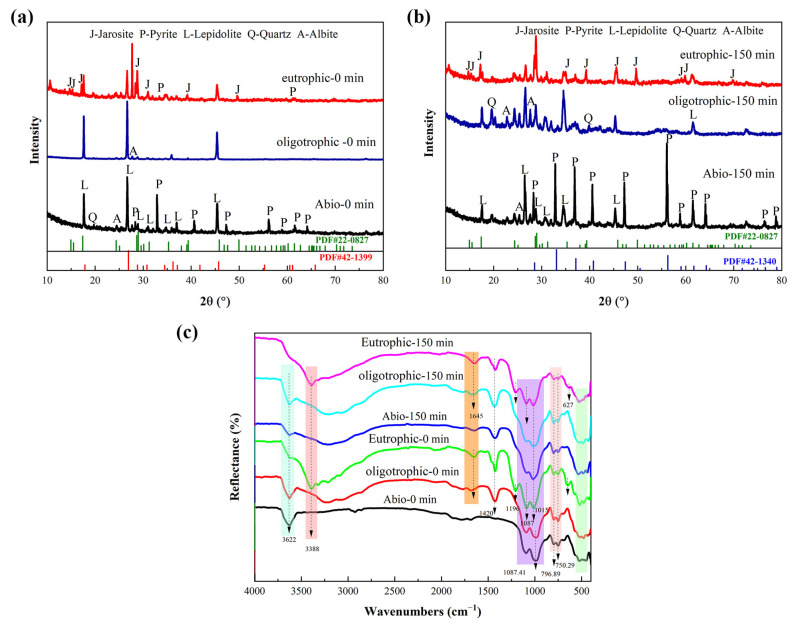
The XRD pattern of solid residues for the nonactivated samples (**a**) and after 150 min of mechanical activation (**b**); FT-IR spectra (**c**) of solid residues for the nonactivated samples and after 150 min of mechanical activation.

The XRD results revealed that the characteristic peak of lepidolite in the solid-phase residue of the sterile control group and oligotrophic bioleaching group before activation was clear and strong. The main phases were lepidolite ([K(Li,Al)_3_(Si,Al)_4_O_10_(F,OH)_2_]), albite ([Na(AlSi_3_)O_8_]), and quartz (SiO_2_) [13]. Under the three leaching conditions, the characteristic peaks of lepidolite significantly decreased with increasing mechanical activation time, whereas the relative intensity of the diffraction peak of aluminosilicate, such as that of celadonite, increased (Figure S4). After bioleaching for 14 days, the characteristic peaks of pyrite in the solid residue of the nonactivated eutrophic groups decreased, and many characteristic peaks of jarosite appeared, which was due to the transformation of the mineral phase under the action of microbial oxidation (Figure 9a). The peak shape of the lepidolite after mechanical activation for 150 min exhibited little change, but the relative intensity of the peak obviously decreased (Figure 9b), and the phase recognition and analysis of the XRD results were consistent with the SEM results (Figure 8). Notably, the change in the characteristic peak of the lepidolite in the leaching residue of the different groups was small because of the existence of high-strength structural units in the structure of the lepidolite minerals, which are difficult to destroy [10].

The leaching residues of the different groups were analyzed by FT-IR, as shown in Figure 9c. The FT-IR spectrum of the leaching residue of the sterile control group was consistent with that of polysilicon lepidolite [10], and there was no significant change in the functional groups. After leaching, the peak attributed to Al-OH bonds in lepidolite at 3362 cm^−1^ decreased in intensity in the bioleaching groups, especially in the eutrophic groups after 150 min of mechanical activation, and the peak at this position completely disappeared, indicating the disruption of metal ion bonds in the lepidolite. In addition, the eutrophic group before and after activation had an absorption peak at 3388 cm^−1^, which was attributed to the stretching vibration of water hydroxyl groups in the interlayer of lepidolite, indicating that amino and hydroxyl groups were involved in the process of metal–bacteria surface binding. The absorption peak of the inverted “mountain” type in the low-frequency range of bioleaching is obviously different from that of the sterile group, which can be attributed to the Li-O and Al-O bonds, which correspond to the release of metal ions in the solution. The characteristic peak at 1450 cm^−1^ represents the stretching peak of COO^−^ [21], and there was an absorption peak near 1645 cm^−1^, indicating that the olefin bonds and carboxyl groups in EPS, which are the main groups of proteins and polysaccharides (the main components of EPS), are involved in the leaching reaction. Schubert et al. [22] reported that iron atoms form bridging compounds by adsorbing -O- or -CO-, which can enhance the transfer of charge carriers between iron ions and minerals, thus increasing the leaching rate. The presence of O-H, C-C, -CH_3,_ and C-H bonds in the metabolites of bioleaching groups enhances the binding strength between bacteria and minerals and plays an important role in the decomposition of lithium silicate ore [20]. The [SiO_4_] stretching vibration band (approximately 1025 cm^−1^) [10] in the leaching residue of the two bioleaching groups was broadened due to the leaching of the aluminosilicate. Furthermore, for the eutrophic group subjected to mechanical activation for 150 min, the characteristic peaks of the SO_4_^2−^ molecular stretching vibration of minerals mainly include two parts [23]. The υ_3_(SO_4_) characteristic peaks were located at 1196 cm^−1^ and 1087 cm^−1^, and the peak at 627 cm^−1^ was the characteristic peak of υ_4_(SO_4_), which was attributed to iron secondary minerals. The results were the same as the XRD and SEM results (Figure 8).

### 3.5. Analysis of the Microbial Community Structure

**Figure 10:**
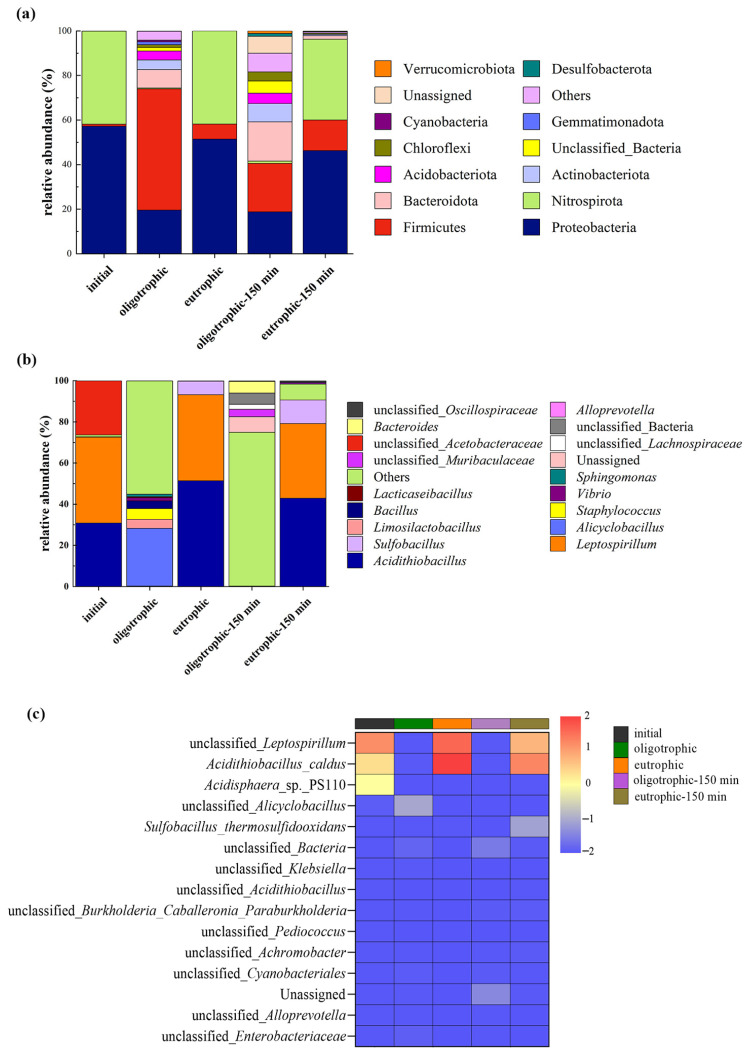
The distribution of the microbial community structure before and after leaching without and with mechanical activation under optimal process conditions: phylum distribution (**a**), genus distribution (**b**), and heatmap at the species level (**c**).

Compared with bioleaching with a single bacterium, bioleaching with a microbial community has more advantages, and the interaction between microbial populations is highly important for improving the efficiency of ore leaching [24]. Variations in chemical and physical conditions during the bioleaching process, such as the pH and the Fe^3+^/Fe^2+^ ratio, are the primary factors that influence microbial dynamics [25]. Analysis of the composition and structural changes in the microbial communities of the eutrophic and oligotrophic groups (nonactivated/activated for 150 min) after 14 days of bioleaching was conducted (Figure 10). Before mechanical activation, the oligotrophic groups were dominated by Firmicutes, with an abundance of 54.4% (Figure 10a). Compared with the abundances before bioleaching, the the abundances of Proteobacteria and Nitrospirota decreased by 37.6% and 41.4%, respectively. Notably, Nitrospirota constituted a mere 0.38% of the total microbial population. In contrast, the eutrophic groups were primarily composed of Proteobacteria (51.4%) and Nitrospirota (41.8%). This might be because environments rich in metal ions provide suitable conditions for the growth of Proteobacteria. Moreover, Nitrospirota utilizes pyrite as an energy source and is acid tolerant; thus, it has a competitive advantage in eutrophic leaching groups, leading to an increase in abundance. On the other hand, some microorganisms belonging to the Firmicutes phylum are sensitive to acidic environments, which makes oligotrophic conditions more favorable for the growth of microorganisms within the Firmicutes phylum. Mechanical activation significantly increased the diversity of microbial communities at the phylum level. After 150 min of activation, significant changes occurred in the proportion of microorganisms at the phylum level in the oligotrophic group. Firmicutes and Proteobacteria accounted for 21.8% and 18.7%, respectively. Although the absolute values of their proportions were relatively small, they were still dominant communities. In comparison, the composition of the microbial communities in the eutrophic group also increased, but with extremely low relative abundances. The relative abundances of Nitrospirota, Proteobacteria, and Firmicutes did not change significantly, but Proteobacteria and Nitrospirota still held absolute dominance. Overall, in both the oligotrophic and eutrophic groups, mechanical activation altered the composition of microbial communities to some extent, which may be related to the adaptability of microorganisms to environmental conditions.

According to the histogram of the relative abundance of microorganisms at the genus level (Figure 10b), before the bioleaching experiment, *Acidithiobacillus*, *Leptospirillum*, and unclassified *Acetobacteraceae* were absolutely dominant. Under oligotrophic conditions, there was a great difference in the diversity of microbial genera before and after leaching, and other acidophilic bacteria (others) were absolutely dominant; however, owing to the lack of sufficient energy substrates such as Fe(II) and FeS_2_, the relative abundances of *Acidithiobacillus* and *Leptospirillium* decreased sharply. The increase in the content of *Alicyclobacillus* to 28% may be because *Alicyclobacillus* can metabolize organic matter produced by other microorganisms; in addition, its own metabolites can promote the leaching of nonsulfide ores, such as silicate ores, through complexation, reduction, acid hydrolysis, and other mechanisms [26]. The effect of pyrite on the leaching bacterial community was more obvious [27]. *Acidithiobacillus* and *Leptospirillium* were the dominant species after leaching under eutrophic conditions, and the relative abundance of *Sulfobacillus* also increased. Mechanical activation also affects microbial communities at the genus level in both oligotrophic and eutrophic groups. Mechanical activation resulted in the continuous dominance of unclassified bacteria in the oligotrophic groups, a significant decrease in the abundance of *Alicyclobacillus*, and the emergence of new genera such as *Vibrio* and *Chloroflexi* with certain abundances after activation. In comparison, in the eutrophic groups, *Leptospirillum* and *Acidithiobacillus* remained the dominant genera both before and after activation. Moreover, there was an increase in *Sulfobacillus* abundance. Furthermore, some new genera appeared for the first time after activation but with relatively low abundances. These results suggest that mechanical activation seems to have a certain reshaping effect on the microbial community structure. The mechanical stress imposed by the activation process likely disrupts the existing microbial equilibrium, favoring the growth of certain taxa and suppressing others. Such perturbations in microbial communities can potentially affect various ecosystem functions and processes.

A heatmap diagram was generated to visualize the microbial sequencing data before and after leaching. According to the gradient and similarity of color, the differences in community composition and species and the similarities of microorganisms at the taxonomic level before and after leaching were determined (Figure 10c) [28]. When the lepidolite was not activated, the relative abundances of unclassified *Leptospirillum*, *Acidithiobacillus caldus*, and *Acidisphaera* sp. PS110 decreased significantly after leaching in the oligotrophic groups, but the opposite trend was observed in the eutrophic groups. The changes in oligotrophic and eutrophic microorganisms before and after mechanical activation were similar at the species level. Notably, after 150 min of mechanical activation, the relative abundance of *Sulfobacillus thermosulfidooxidans* significantly increased in the eutrophic leaching groups. These results indicate that for the bioleaching processes of lepidolite, mechanical activation increases the diversity of the microbial community, which helps to improve the overall function of the microbial community, enhances adaptability to different environmental conditions, increases the overall metabolic efficiency, and thus increases the leaching rate of ions.

### 3.6. A Discussion of the Leaching Rate and Leaching Mechanism

**Figure 11:**
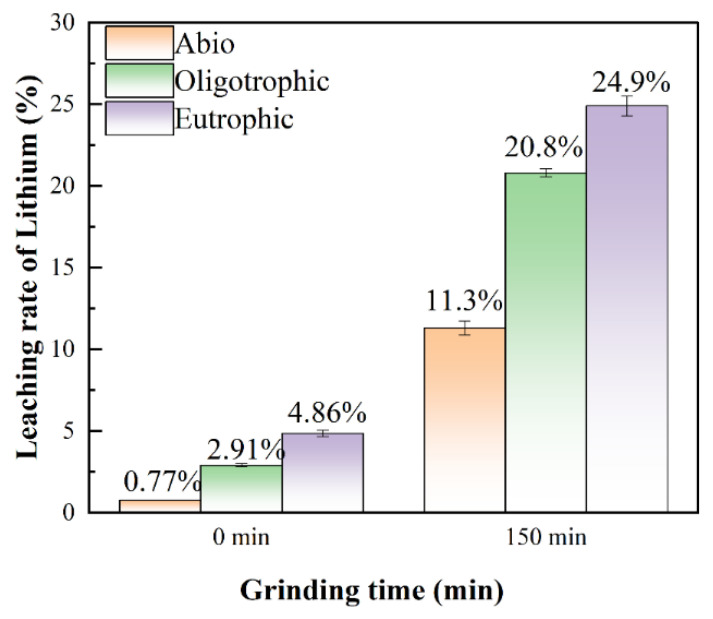
The leaching rates of lithium for the sterile control, oligotrophic, and eutrophic groups without mechanical activation and after 150 min of mechanical activation.

In this study, it was found that the reaction activity of ions in lepidolite can be greatly improved by mechanochemical activation combined with interactions between microorganisms and minerals. This could be attributed to the fact that after mechanical activation, microorganisms are more likely to penetrate into the depths of these lepidolite craters and cracks and continue to erode the ore [29]. Generally, the recovery of lithium is mainly concentrated in the leaching solution. After 14 days of leaching, the leaching rates of lithium in the sterile control group and oligotrophic and eutrophic bioleaching groups after activation for 150 min were 11.3%, 20.8%, and 24.9%, respectively, which were 10.53%, 17.89%, and 20.04% greater than those without activation (Figure 11). The leaching rates of aluminum ions before and after mechanical activation are presented in Figure S5. In addition, regardless of whether the lepidolite was activated, eutrophic bioleaching improved the leaching rate of the elements in the lepidolite. Notably, under the experimental conditions of mechanical activation, although the leaching rate was still relatively low, the leaching rate of lithium under the experimental conditions in this study significantly improved compared with that in the literature, approximately 3 times greater than that reported by Sedlakova-Kadukova et al. [7], and the leaching time (14 d) was shorter than that (41 d), indicating great application potential. Moreover, Kirk et al. [30] recently reported that the bioleaching efficiency of lithium from lepidolite using *Acidiothiobacillus ferrooxidans* was only 14% within 30 days. In addition, the current mainstream lithium extraction technology for lepidolite is the sulfate roasting method. Its advantages are a relatively high recovery rate (approximately 75%) and the ability to process low-grade lepidolite. The disadvantages are the high cost of the raw material potassium sulfate, long process flow, relatively large amount of slag, and higher comprehensive costs than those of salt lakes and spodumene, which are approximately CNY 60,000–80,000 per ton in China. In this work, the bioleaching method eliminated the need to consider issues such as waste residue treatment, equipment corrosion and maintenance, requirements for other materials, and subsequent environmental remediation. This seemingly results in substantial cost savings. Compared with processes involving strong acids, strong alkalis, and high-temperature roasting, this method reduces both energy consumption and costs while being environmentally friendly. Furthermore, although the bioleaching cycle is relatively long, employing mechanical activation as a pretreatment effectively enhances the extraction efficiency of elements from lepidolite.

**Figure 12:**
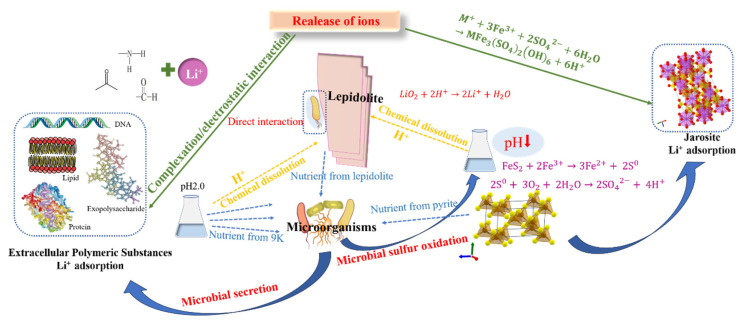
A proposed diagram of the mechanism of lepidolite bioleaching after mechanical activation.

The mechanical activation pretreatment process can enhance the disruption of Si-O-K and Si-O-Li structures within the lepidolite laminar structure, thereby reducing the bond strength of Li-O [11]. Meanwhile, the average particle size of lepidolite is significantly reduced, and the specific surface area is increased. Moreover, during the subsequent bioleaching process, more cracks and pores are generated in lepidolite, which enlarges the contact area between microorganisms and minerals, providing more favorable conditions for growth and metabolic activities and thus effectively increasing the bioleaching efficiency. Additionally, bioleaching under mechanical activation increases the diversity of the microbial community, thereby positively affecting the increase in the lithium leaching rate. On the basis of the above analysis, a schematic diagram of the leaching process of lepidolite with different interaction groups was proposed, as shown in Figure 12. The conventional leaching of Li generally uses a large amount of protonic acid to enhance the decomposition of silicon–oxygen tetrahedrons in lamellar structures, resulting in the dislocation of stable Si-O-Li structures, increased Li-O activity, and efficient leaching of Li between lamellar structures [11]. Regardless of whether mechanical activation is performed before leaching, owing to the initial acidic environment, different leaching groups can slowly promote leaching. The presence of microorganisms significantly promotes the leaching of elements from lepidolite. On the one hand, silicate bacteria and autotrophic bacteria in the microbial community may adsorb onto the surface of lepidolite, playing a direct role in leaching. On the other hand, the extracellular polymeric substances produced by microorganisms can adsorb positively charged lithium ions through electrostatic or complexation interactions. Although there is no pyrite energy substrate in oligotrophic groups, microorganisms are forced to obtain nutrients needed for life activities directly from lepidolite, thus promoting leaching; in eutrophic bioleaching groups, acidophilic microorganisms oxidize pyrite, and the subsequent hydrolysis of Fe^3+^ in the solution releases H^+^. The protons enhance the decomposition of silicon–oxygen tetrahedrons in lamellar structures, resulting in the dislocation of stable Si-O-Li structures, increased Li-O activity, and the efficient leaching of Li between lamellar structures [11], and separating metal ions from ore particles by reducing their binding strength, enhancing the mobility of metal ions and promoting their leaching from minerals. Additionally, secondary minerals form in eutrophic bioleaching groups because their high iron content can also adsorb lithium ions. The adsorption of lithium by cells and secondary minerals greatly contributes to the dissolution of lepidolite, thus improving the leaching efficiency of lithium. This is due to the rapid accumulation of lithium ions released in the solution on the cell surface and in secondary minerals, which can cause the dissolution equilibrium of lepidolites to shift in the direction of Li^+^ ion release. Consequently, in this study, pretreatment of lepidolite with mechanical activation prior to leaching effectively improved the leaching efficiency of the tested elements.

## 4. Conclusions

The use of mechanical activation as a pretreatment for lepidolite followed by bioleaching technology can increase the lithium recovery rate from lepidolite. With a rotation speed of 200 r/min, a mineral-to-ball ratio of 1:20, and activation for 150 min, the lithium leaching rates were 11.3%, 16.75%, and 19.24% after 14 days of leaching for the sterile control and the oligotrophic and eutrophic groups, respectively. Owing to the initial acidic environment, lepidolite slowly leached into the sterile control groups, whereas the presence of acidophilic microorganism communities in the presence or absence of pyrite significantly enhanced the reaction kinetics of Li^+^ leaching in the groups with activated lepidolite. Notably, mechanical activation increased the diversity of the microbial community in the bioleaching groups. Compared with the reported bioleaching process for lepidolite, the leaching rate of lepidolite in this study significantly improved with a shorter leaching time, demonstrating great potential for application in the field of lithium extraction from hard rock ores.

## Data Availability

The original contributions presented in the study are included in the article material; further inquiries can be directed to the corresponding author.

## Supplementary Materials

The following supporting information can be downloaded at https://www.mdpi.com/article/10.3390/microorganisms13020415/s1. Figure S1: Basic procedure of the leaching experiment; Figure S2: Chromogenic reaction of polysaccharides in the phenol-sulfuric acid assay in the (a) oligotrophic group and (b) eutrophic group following 150 min of mechanical activation; Figure S3: SEM morphology after leaching following 120 min of mechanical activation for the (a) sterile group, (b) oligotrophic group, and (c) eutrophic group; Figure S4: SEM-EDS images of eutrophic systems showing (a) non-activated products and (b) products after 150 min of mechanical activation; Figure S5: XRD patterns after leaching in the (a) sterile group, (b) oligotrophic group, and (c) eutrophic group; Figure S6: The leaching rate of aluminum (non-activation/activation for 150 min); Table S1: Chemical composition of the raw ore of lepidoptera (in terms of oxide content and mass fraction); Table S2: The absorbance of orange-yellow derivatives generated by the phenol-sulfuric acid assay; Table S3: The concentration of lithium ions in extracellular polymeric substances.

## References

[1] Chen S.B., Xu C., Yan X.X., Chen X., Xie Y.C. (2022). Research progress on lithium extraction from ores and salt lakes. New Energy Technol..

[2] Yu Y.S., Cui L.X., Wang Y.F., Zhang L.B. (2022). Research progress of lithium extraction technology from lepidolite. Chin. J. Nonferrous Met..

[3] Su H., Zhu Z.W., Wang L.N., Qi T. (2019). Research progress in extraction and recovery of lithium from hard-rock ores. CIESC J..

[4] Rezza I., Salinas E., Calvente V., Benuzzi D., de Tosetti M.I.S. (1997). Extraction of lithium from spodumene by bioleaching. Lett. Appl. Microbiol..

[5] Marcincakova R., Kadukova J., Mrazikova A. (2015). Bioleaching of Lithium from Lepidolite by the Mixture of *Rhodotorula rubra* and *Acidithiobacillus ferrooxidans*. Inz. Miner. J. Pol. Miner. Eng. Soc..

[6] Reichel S., Aubel T., Patzig A., Janneck E., Martin M. (2017). Lithium recovery from lithium-containing micas using sulfur oxidizing microorganisms. Miner. Eng..

[7] Sedlakova-Kadukova J., Marcincakova R., Luptakova A., Vojtko M., Fujda M., Pristas P. (2020). Comparison of three different bioleaching systems for Li recovery from lepidolite. Sci. Rep..

[8] Luong V.T., Kang D.J., An J.W., Kim M.J., Tam T. (2013). Factors affecting the extraction of lithium from lepidolite. Hydrometallurgy.

[9] Alex T.C., Kumar R., Roy S.K., Mehrotra S.P. (2016). Mechanical Activation of Al-oxyhydroxide Minerals-A Review. Miner. Process. Extr. Metall. Rev..

[10] Vieceli N., Nogueira C.A., Pereira M.F.C., Dias A.P.S., Durao F.O., Guimaraes C., Margarido F. (2017). Effects of mechanical activation on lithium extraction from a lepidolite ore concentrate. Miner. Eng..

[11] He M.M., You H.X., Zhao C.L., Zheng X.B., Cao H.B., Sun Z. (2019). Technology of lithium extraction from lepidolite through mechanochemistry activation. Chin. J. Process Eng..

[12] Beiranvand Z., Ahmadi A., Hosseini M.R. (2023). Effect of mechanical activation on biooxidation and gold extraction of a high-grade flotation concentrate using mesophilic and moderately thermophilic microorganisms. Miner. Eng..

[13] Yan Q.X., Li X.H., Wang Z.X., Wang J.X., Guo H.J., Hu Q.Y., Peng W.J., Wu X.F. (2012). Extraction of lithium from lepidolite using chlorination roasting-water leaching process. Trans. Nonferrous Met. Soc. China.

[14] Ma P.P., Yang H.Y., Luan Z.C., Sun Q.F., Ali A., Tong L.L. (2021). Leaching of Chalcopyrite under Bacteria-Mineral Contact/Noncontact Leaching Model. Minerals.

[15] Norlund K.I., Baron C., Warren L. (2010). Jarosite formation by an AMD sulphide-oxidizing environmental enrichment: Implications for biomarkers on Mars. Chem. Geol..

[16] Lv Y., Li J., Ye H.P., Du D.Y., Li J.X., Sun P., Ma M.Y., Wen J.X. (2019). Bioleaching behaviors of silicon and metals in electrolytic manganese residue using silicate bacteria. J. Clean. Prod..

[17] Zhang Y., Zheng S.L., Du H., Xu H.B., Zhang Y. (2010). Effect of mechanical activation on alkali leaching of chromite ore. Trans. Nonferrous Met. Soc. China.

[18] Wang Y.J., Yuan X.Q., Fu H.Q. (2014). Mineralogical Characteristics of Gem-quality Lepidolite Rock in Xinjiang. J. Gems Gemmol..

[19] Gao T.M., Fan N., Chen W., Dai T. (2023). Lithium extraction from hard rock lithium ores (spodumene, lepidolite, zinnwaldite, petalite): Technology, resources, environment and cost. China Geol..

[20] Zhao X., Zhou Y., Ding C., Wang X., Zhang X., Wang R., Lu X. (2023). Lithium extraction from typical lithium silicate ores by two bacteria with different metabolic characteristics: Experiments, mechanism and significance. J. Environ. Manag..

[21] Liu J.S., Wang Z.H., Li B.M., Zhang Y.H. (2006). Interaction between pyrite and cysteine. Trans. Nonferrous Met. Soc. China.

[22] Schubert B., Tributsch H. (1990). Photoinduced electron transfer by coordination chemical pathways across pyrite/electrolyte interfaces. Inorg. Chem..

[23] Chen L., Wang Y.R., Liu H.C., Zhou Y.H., Nie Z.Y., Xia J.L., Shu W.S. (2025). Different fates of Sb(III) and Sb(V) during the formation of jarosite mediated by *Acidithiobacillus ferrooxidans*. J. Environ. Sci..

[24] Ma X.M., Han Y.F., Lu F.P., Zhang X.X., Wen D., Liu Y.J., Bai L.M., Hu Y., Huang Z.Y. (2012). Study of coupled pyrite leached pyrolusite by functional microflora. Microbiol. China.

[25] Han Y.F., Ma X.M., Zhao W., Chang Y.K., Zhang X.X., Wang X.B., Wang J.J., Huang Z.Y. (2013). Sulfur-oxidizing bacteria dominate the microbial diversity shift during the pyrite and low-grade pyrolusite bioleaching process. J. Biosci. Bioeng..

[26] Meng C.Y., Liu W.Y., Liu X.Y., Chen B.W., Wen J.K. (2015). Review on the genus *Alicyclobacillus* and the study on their biohydrmetallurgy APPLICATION. Min. Metall..

[27] Zheng H.A., Zhang C., Wu Y., Shi P.H., Wang Z.H., Wang X.J., Li B.Y., Liu J.S., Xie X.H. (2015). Bioleaching of arsenic-containing gold ore influenced by cysteine. Fresenius Environ. Bull..

[28] Mallappa R.H., Singh D.K., Rokana N., Pradhan D., Batish V.K., Grover S. (2019). Screening and selection of probiotic *Lactobacillus* strains of Indian gut origin based on assessment of desired probiotic attributes combined with principal component and heatmap analysis. LWT Food Sci. Technol..

[29] Nkulu G., Gaydardzhiev S., Mwema E., Compere P. (2015). SEM and EDS observations of carrollite bioleaching with a mixed culture of acidophilic bacteria. Miner. Eng..

[30] Kirk R.D., Newsome L., Falagan C., Hudson-Edwards K.A. (2024). Bioleaching of lithium from jadarite, spodumene, and lepidolite using *Acidiothiobacillus ferrooxidans*. Front. Microbiol..

