# Supplementary material for: Enhanced Leaching of Lepidolite by Acidophilic Microorganisms Under Mechanical Activation
Source: Microorganisms. 2025 Feb 13;13(2):415. doi: 10.3390/microorganisms13020415 (PMC11858582; doi:10.3390/microorganisms13020415)
Supplement: Supplementary file 1 [file microorganisms-13-00415-s001.zip › microorganisms-3462615-supplementary.pdf]

Supplementary Information

for

Enhanced leaching of lepidolite by acidophilic microorganisms under mechanical activation

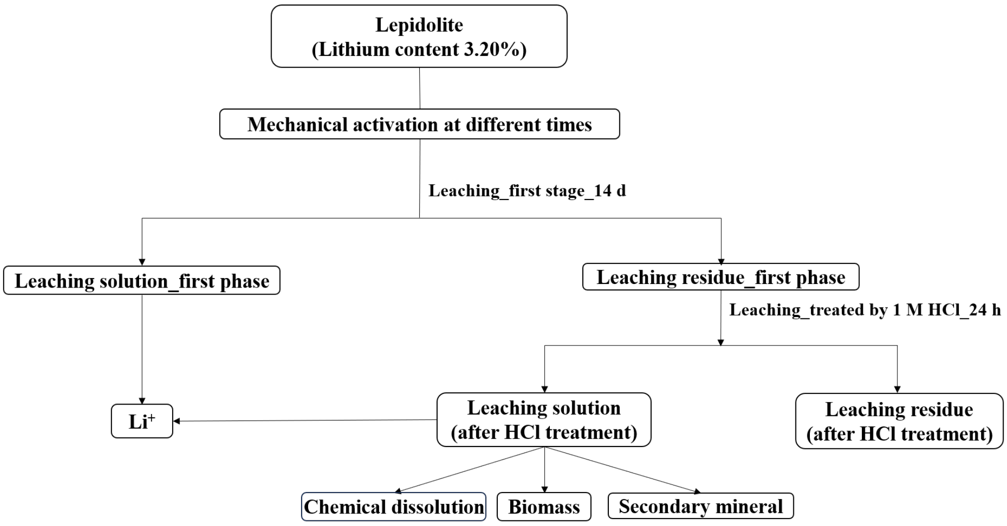

Figure S1 Basic procedure of the leaching experiment.

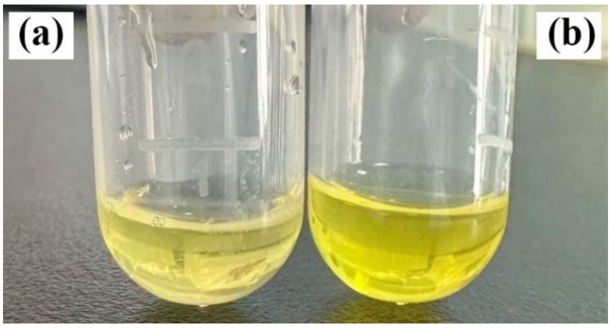

Figure S2 Chromogenic reaction of polysaccharides in the phenol-sulfuric acid assay in the (a) oligotrophic group and (b) eutrophic group following 150 minutes of mechanical activation

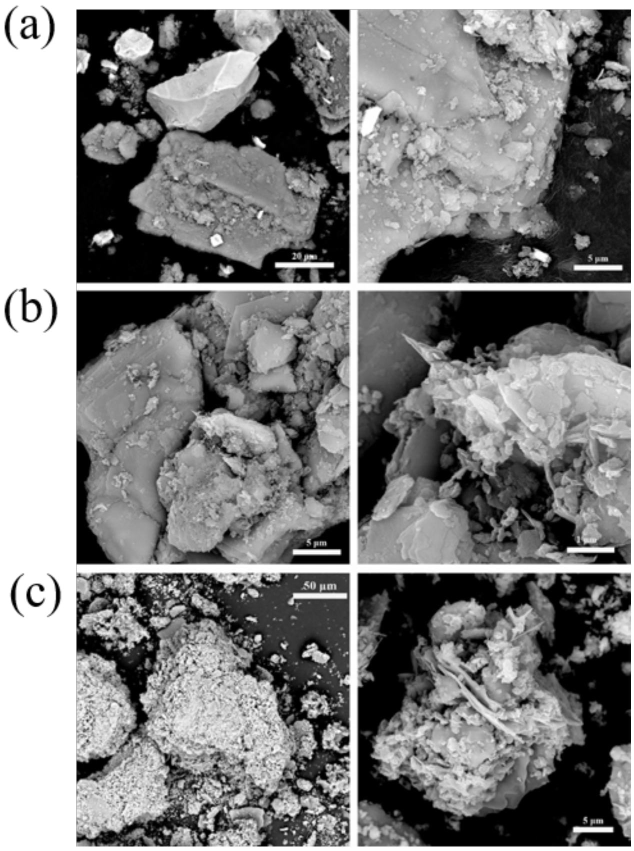

Figure S3 SEM morphology after leaching following 120 minutes of mechanical activation for the (a) sterile group, (b) oligotrophic group, and (c) eutrophic group.

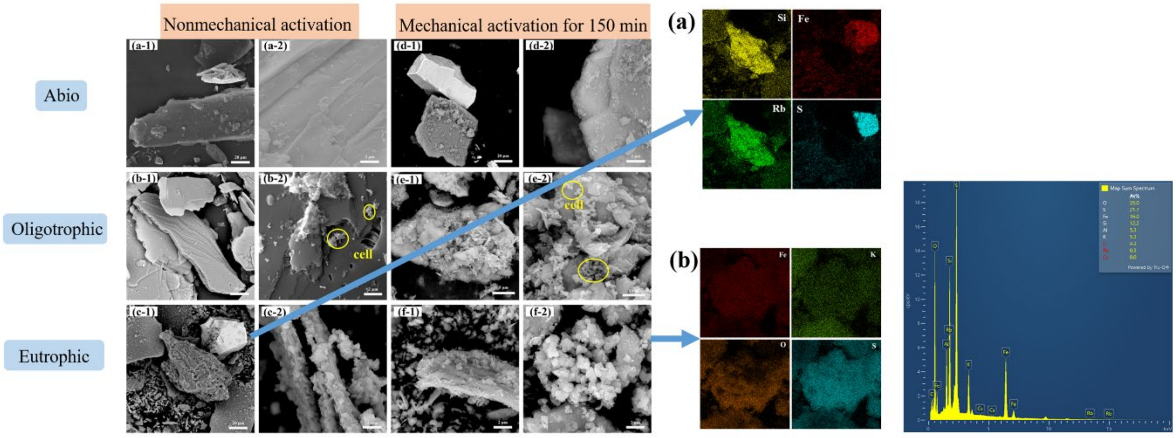

Figure S4 SEM-EDS images of eutrophic systems showing (a) non-activated products and (b) products after 150 minutes of mechanical activation.

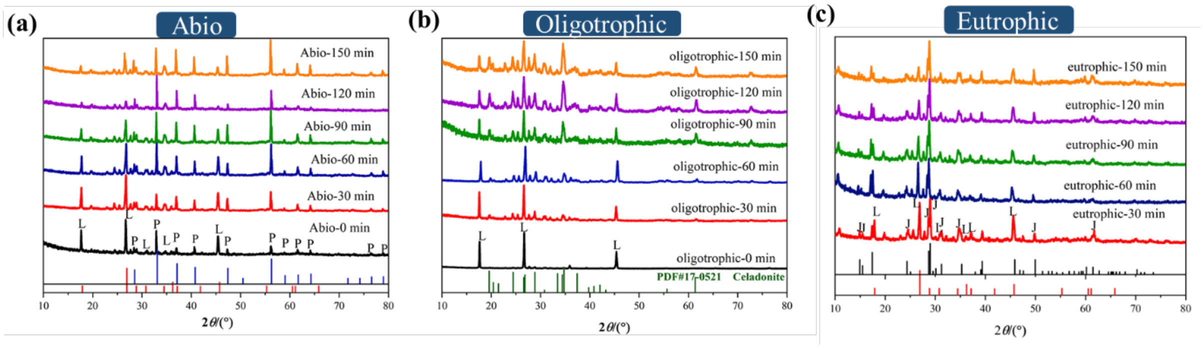

Figure S5 XRD patterns after leaching in the (a) sterile group, (b) oligotrophic group, and (c) eutrophic group.

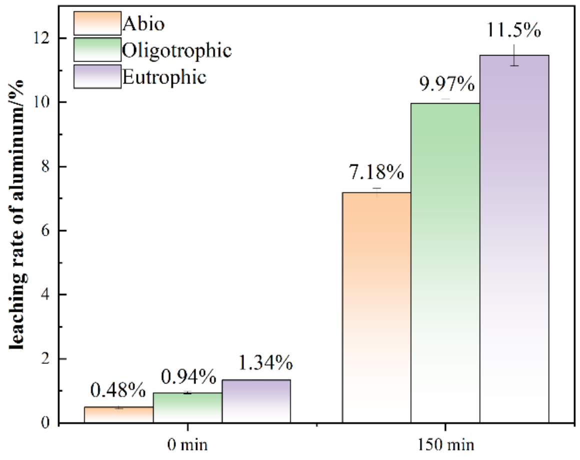

Figure S6 The leaching rate of aluminum (non-activation/activation for 150 minutes)

Table S1 Chemical composition of the raw ore of lepidoptera (in terms of oxide content and mass fraction)

Na_2O	Al_2O_3	SiO_2	K_2O	Mn_2O_3	SO_3
0.28	26.35	55.22	11.26	1.36	0.44

Table S2 The absorbance of orange-yellow derivatives generated by the phenol-sulfuric acid assay

**Bioleaching group**	**The spectrophotometric absorbance (490 nm)**
Oligotrophic system with mechanical activation for 150 minutes	0.294
Eutrophic system with mechanical activation for 150 minutes	0.656

Table S3 The concentration of lithium ions in extracellular polymeric substances

**Bioleaching group**	**Lithium ion concentration (μg/L)**
Oligotrophic system with mechanical activation for 150 minutes	1.13
Eutrophic system with mechanical activation for 150 minutes	3.83
